# Binge-type eating disorders and ultra-processed food addiction: phenomenology, pathophysiology and treatment implications

**DOI:** 10.3389/fpsyt.2025.1584891

**Published:** 2025-06-20

**Authors:** Joan Ifland, Timothy D. Brewerton

**Affiliations:** ^1^ Food Addiction Reset LLC, Seattle, WA, United States; ^2^ Department of Psychiatry and Behavioral Sciences, Medical University of South Carolina, Charleston, SC, United States

**Keywords:** food addiction, binge eating, ultra-processed food, eating disorders, loss of control, bulimia, anorexia, purging

## Abstract

**Background and objective:**

Despite their clinical differences, loss of control binge eating (LCBE) is a core feature of all binge-type eating disorders (EDs), including binge eating disorder (BED), bulimia nervosa (BN), and anorexia nervosa binge purge type (AN-BP). The emerging concept of food addiction (FA), or ultra-processed food addiction (UPFA), is also characterized by LCBE. However, LCBE treatment has rejected addiction recovery approaches, especially abstinence or reduced harm through reduced use, to the detriment of patients. Treatment could be more successful if barriers to addiction recovery protocols such as reduced harm and abstinence were addressed.

**Hypothesis and theory:**

The phenomenology and clinical features of binge-type EDs and UPFA overlap considerably, yet they also have distinct clinical features and treatment approaches. Among their commonalities, these conditions share pathophysiological mechanisms. Specifically, available evidence demonstrates that LCBE, regardless of diagnosis, is characterized by alterations in neurobiological systems mediating reward sensitivity, stress reactivity, and cognitive function that are similar to the disturbances found in Ultra-Processed Food Addiction (UPFA), Alcohol Use Disorder (AUD) and other substance use disorders (SUDs). Ultra-processed foods (UPFs) used by patients with LCBE have clearly been shown to have powerful addictive properties. However, the key substance use disorder (SUD) recovery protocols of harm reduction or abstinence from addictive substances are not commonly employed in the treatment of binge-type EDs. The objectives of this paper are to organize evidence that the LCBE characteristic of binge-type EDs and UPFA overlap in many cases and to consider the impact of these findings on treatment protocols, specifically the application of harm reduction and/or abstinence from psychoactive UPFs. This hypothesis can be tested in clinical trials of individuals with LCBE.

**Results:**

Neurobiological studies of individuals with LCBE consistently show signs of addictive alterations, especially hyperactive reward centers, stress reactivity, and cognitive impairment, as well as maladaptive use of UPFs. This is very similar to the results of addictive use of alcohol for which abstinence and harm reduction are demonstratively helpful. However, this approach has not been used in the eating disorders field which may be to the detriment of patients with LCBE.

**Discussion:**

These findings suggest that treatment outcomes for binge-type EDs characterized by LCBE might improve if harm reduction and/or abstinence protocols for recovery from UPFA were applied. A level of support high enough for a severe addiction could improve treatment outcomes for these often recurrent and treatment refractory disorders. Possible rationales for current treatment exclusion or marked reduction of UPF abstinence protocols are offered.

## Introduction

Approximately 17% of people worldwide suffer from broadly defined eating disorders (EDs) (1). The great majority of EDs in the general population are binge-type EDs, which are characterized by loss of control binge eating (LCBE), and include binge eating disorder (BED), bulimia nervosa (BN), and anorexia nervosa binge-purge type (AN-BP), as well as subthreshold forms of BN and BED characterized as forms of other feeding and eating disorder (OSFED) (2–5). LCBE is defined by the DSM 5 as consuming a large amount of food in a short period (typically within two hours) while experiencing a subjective sense of inability to stop eating or regulate the type of quantity of food consumed (6). LCBE has been thought to be a core component of objective binge eating. This is in contrast to food addiction which can be characterized by the 11 DSM 5 criteria used for Alcohol Use Disorder (AUD) and other SUD. This is discussed in more detail below. Eating disorders are typically treated with a combination of psychotherapy, medical care, nutritional counseling, and psychotropic medications (7, 8). However, the overall rate of recovery for all ED patients pooled together is only 46% with a mean follow-up interval of 45 months (9).

Over the last several years, evidence has accumulated that supports a diagnosis of addiction to processed or ultra-processed foods (UPFs). Ultra-processed food addiction (UPFA) is also characterized by LCBE and phenomenologically overlaps with diagnoses of all of binge-type EDs (10–22). Further, brain studies of people with obesity show neuroadaptations that parallel those of people with AUD as well as patients with LCBE. These consist of hyperactive reward centers, hyperactive stress pathways, and hypoactive cognitive functions (23). As might be expected, UPFs are shown to be associated with weight gain. In a study of humans in a controlled laboratory setting, Hall at al. found that a diet that contained ultra-processed foods resulted in greater weight gain than a diet of unprocessed foods (24). Processed foods are found to be associated with addictive eating (25). These findings reinforce LaFata and Gearhardt’s analysis of the correlation of the development of obesity with the rise in consumption of UPF (26). The importance of distinguishing between weight status and LCBE or UPFA is illustrated by Wiss et al. who found an increased prevalence of food addiction among an underweight population (29). This reinforces the value of treating based on factors other than weight status. The evidence demonstrates that obesity shows neuro-adaptations in addictive patterns similar to both AUD and binge-type eating disorders.

These overlapping mechanisms suggest a possible framework that could lead to improved results in the treatment of LCBE. Methods consistent with recovery from substance use disorders (SUD) as applied to addictive ultra-processed foods could be effective in the treatment of binge-type EDs. Such approaches could include the use of harm reduction that builds to abstinence under a high level of support consistent with a severe addiction (27). The essence of the concept of harm reduction is to ameliorate adverse consequences of drug use while, at least in the short term, drug use continues (28). Harm reduction is as opposed to abstinence which has the goal of not using the drug at all. These concepts have been adapted to use in recovery from addictive foods.

The reduced harm/abstinence approach is supported by research showing addictive properties for UPFs that are found in binge-type EDs such as sugar (29), high fructose corn syrup (30), flour (31), gluten (32), salt (33), dairy (34), excessive fat (35), caffeine (36), and food additives (37). Research has shown that individuals with LCBE almost exclusively binge on UPFs (38).

The addictive properties of UPFs could explain the addictive neuroadaptations found in populations that eat these processed foods, including populations with binge-type EDs. This approach is supported by numerous similarities in syndromes between people with obesity, who have been shown to be high consumers of UPFs, and people with alcohol/drug addiction. On a macro level, business practices of the tobacco and processed food industries are similar (39). Subsidies for tobacco and for wheat, corn, and sugar keep the prices of the products low enough to make them affordable enough to be used often enough to keep the addiction active (40). The overlap between alcohol/drug and UPFA is consistent, which supports a role for SUD recovery approaches to binge-type EDs, including abstinence and harm reduction strategies.

Our perspective is that individuals diagnosed with a binge-type ED characterized by LCBE are likely experiencing UPFA and are often being treated with protocols that are not consistent with SUD recovery, specifically protocols that exclude abstinence and harm reduction strategies. We argue that this could be the case regardless of etiology. Whether the addiction started from a single origin of repeat exposure to the addictive processed foods found pervasively in western diets, or whether the addiction was exacerbated by a drive to numb trauma, or whether bingeing developed in response to calorie restriction (dieting), the results are the same, i.e. that the brains of people with LCBE diagnoses exhibit the signs of SUD. These findings support the argument that using addiction recovery protocols of reduced harm and abstinence could be valuable regardless of the factors fostering the addiction. As with any SUD recovery program, behavioral habits and the effects of trauma may need to be addressed alongside abstinence or reduced harm.

## Limitations

Substance addictions and eating disorders are vast fields. This paper is focused specifically on key similarities of LCBE and UPFA versus dissimilarities in treatment protocols related to processed food use of reduced harm and abstinence. Thus, this paper does not address such issues as the etiology and development of addictive or eating disorders. It does not address the consequences of overconsumption of processed foods with the exception of obesity. It also does not address treatment outside the issue under consideration which is the absence of reduced harm and abstinence from processed foods in treatment of LCBE. For readers who wish to pursue topics outside the scope of this paper, we recommend the following. For in-depth understanding of the neurology of substance use disorders and hedonic eating, we refer readers to Chapters 7 (41) and 8 (42) of Wilcox 2021. For better understanding of other SUD treatment approaches, Val-Laillet et al. would be helpful (43). To better understand the etiology and development of SUD and LCBE, we recommend Kwako et al. (44).

## Hypothesis and theory

Although binge-type EDs characterized by LCBE are phenomenologically similar to UPFA, as defined by various versions of the Yale Food Addiction Scale (YFAS), their respective treatments may involve very different approaches, particularly when it comes to the question of nutritional therapy (45–47). This paper focuses on the prominent characteristics of addiction which are also found in binge-type EDs or LCBE: alterations in reward sensitivity, cognitive impairment, and stress responding (48). Most review articles found several of these addictive neuroadaptations in LCBE populations, some of which involved UPFs.

This paper stands on specific studies showing the neurological similarities between LCBE and UPFA. As shown below, the studies are numerous, varied in design, and consistent in results.

### Reward alterations

Neuroimaging research shows consistent alterations in reward pathways of individuals with binge-type EDs. Skunde et al. found diminished frontostriatal brain activation in patients with BN vs those without (49). In a review article, Frank found that dopamine pathways were hyporesponsive in bulimia and obesity consistent with downregulation found in drug-addicted populations (50). In a review of 58 brain imaging studies of people with BED, Leenaerts et al. found systematic structural and functional changes in the reward system (51). In a review of animal studies, Blanco-Gandia and colleagues found that palatable foods lead to reward sensitization which can support the development of drug addiction (52). In a second study, these investigators recommended that nutritional patterns be used in treatment of substance use disorders (53). Reward alterations are also consistently found in research on binge eating. In a review article, Hadad and Knackstedt found that BN shared increased dopamine production similarly to drug addiction (54). Lee et al. describes reward sensitivity that is found in both BE and BN regardless of differences in other neuroalterations between the two conditions (55). Simon et al. found that patients with binge-eating and bulimia showed a greater response to food rewards compared to healthy controls. The higher response was related to higher levels of trait food craving, and external eating (56). In a review of animal studies of neurochemical alterations in binge-eating, Avena et al. found that alterations in dopamine (DA), acetylcholine (ACh) and opioid systems in reward-related brain areas occur in response to binge eating of palatable foods (57). In a systematic review of reward sensitivity and eating behavior, Sutton et al. found that reward sensitivity is highly correlated with emotional and binge eating (58). In a review of LCBE populations, cannabinoid systems were dysregulated in a manner consistent with addiction (59). Schag et al. found that reward sensitivity was higher in an obese population with BED than an obese population without (60). Boguzs et al. found that AUD was 1.5 times more likely in a binge-eating population than a control group suggesting reward sensitization in LCBE (61).

These review articles show a consistent pattern of dysregulated reward systems which is characteristic of SUDs such as drug and alcohol addiction as well as addiction to UPFs (62).

### Cognitive impairment

Aloi et al. used neurological tests to find lack of attention and poor decision-making in a population with LCBE (63). In a review article of fMRI studies of disinhibited eating, Giddens et al. found that stress deactivated regions implicated in cognitive control (64). These findings are also consistent with findings in drug addiction (65).

### Both reward alterations and cognitive impairment

In a review of weight-loss and eating disorder literature, Brassard and Balodis found that LCBE populations demonstrated greater decision-making impairments and greater wanting (or craving) for high-fat sweet foods (66). Giel et al. found that BED represents a distinct phenotype within the obesity spectrum that is characterized by increased impulsivity and reward sensitivity specifically towards food (67). In a narrative review, Hartogsv et al. describe the three alterations most prominent in binge-type EDs as increased reward sensitivity, decreased cognitive control, and altered stress responding (48). Kessler et al. synthesized neuroimaging, neuro cognitive, genetics, and animal studies to find an impulsive/compulsive disorder with altered reward sensitivity and food-related attentional biases which are similar to alterations observed in substance abuse (68). In a review of 100 publications of the phenomenology of neuroendocrine changes, emotional homeostasis factors, and reward circuits published between 2000 and 2021, Milano et al. found that these phenomenon are associated with exposure to highly palatable foods, loss of control, the way we eat, an increase in impulsiveness and the inability to change eating behavior despite the negative consequences related to overweight and obesity (23).

### Stress

Stress, trauma and its effects, including the development of Post Traumatic Stress Disorder (PTSD) and related comorbidity, has been shown to be a prominent clinical characteristic associated with both UPFA and LCBE (69–82). Chronic stress administered to animals increases susceptibility to binge-eating and food addiction (83). Similarly, stress and associated negative affect, is one of the most common triggers of binge-eating (84). Sinha found in a review article that UPF stimulate both reward and stress circuitry contributing to cravings, excessive food intake, and weight gain (85).

Consumption of UPFs simultaneously produces a combination of pleasurable reinforcing effects and reinforcing “comforting” effects that in the short term normalize an individual’s responses to stress, yet repetitive and intermittent UPF intake may instead amplify brain stress circuitry and downregulate the brain’s reward pathways in such a way that continued intake becomes obligatory to prevent the development of negative emotional states via negative reinforcement (86). This interplay between stress/trauma, hedonic reward and affective dysregulation helps to explain why patients with LCBE/UPFA have significantly higher psychopathology and lower quality of life (71, 72, 87, 88 ).

### Involvement of ultra-processed foods

Senol et al. reviewed research on the dysregulated pathways found in EDs and found that excessive consumption of energy-dense foods alters the brain circuits implicated in reward, decision-making, control, habit formation, and emotions that are central to drug addiction (89). Steward et al., in a review of fMRI studies of people with EDs found heightened responses to food cues and anticipated food receipt occurring with diminished recruitment of cognitive control circuitry which combine to contribute to LCBE of palatable foods (90). Vasiliu et al. found evidence for an overlap between food addiction and EDs to the point of questioning whether they are distinct diagnoses (91). However, the article only mentions pharmaceuticals for therapeutic solutions. In a narrative review, Via et al. connected UPFs with poor impulse control, hyperactivity of reward regions and precedents for subthreshold BED and BED in a population of children and adolescents (92). Vrieze et al. assert that understanding the specific underlying aberrant reward mechanisms in LCBE, associated with different stages of the illness, enables caregivers to focus their treatment more precisely (93).

### Comparison of alcohol use disorder, ultra-processed food addiction, and binge-type eating disorders in terms of approaches to abstinence

To compare and summarize the evidence, the **Table 1** shows similarities in the characteristics of AUD, UPFA, and LCBE while contrasting their respective approaches to treatment. AUD was chosen for the comparison because it is an established addictive substance that, like UPFs, also has calories and is legal.

**Table 1:** Characteristics of alcohol use disorder, ultra-processed food addiction, and binge-type eating disorder vs approaches to abstinence in treatment.

Characteristic	Alcohol use disorder	Ultra-processed food addiction	Binge-type eating disorders
Dopamine dysregulation	Altered reward processing, complex conditioning, impaired learning, and increased reaction to cues (94)	Repeated release of dopamine following overeating of sugar/fat (95)	The neurotransmitter dopamine is involved in food craving, decision making, executive functioning, and impulsivity personality trait; all of which contribute to the development and maintenance of binge eating (62)
Opioid dysregulation	Opioids are released following consumption of alcohol (96)	Opioid-like withdrawal from sugar/fat (95)	Opioid-mediated hedonic and motivation processes drive disorders of ‘appetitive motivation’ including binge-eating disorder (97)
Cannabinoid dysregulation	Alcohol disturbs cannabinoid pathways and increases sensitivity to alcohol signals (98)	A large body of evidence supports the involvement of the cannabinoid system in food addiction (99)	The review highlights the specific role of the endocannabinoid system in the development and maintenance of BED (59)
Activation of stress	Dynorphin and orexin are activated (100)	Stress increases susceptibility to food addiction (83)	Stress is one of the most common triggers of binge-eating (84)
Cognitive impairment	AUD is associated with cognitive impairment (101)	A significant overall effect suggests that individuals with food addiction have poorer performances when completing cognitive tasks (102)	Individuals with BED showed poorer performances at tasks assessing cognitive flexibility, inhibitory control, attention and planning (102)
Treatment Approaches related to Abstinence	A degree of abstinence is desirable for recovery of control (103)	From refined carbohydrates (104–106)	Abstinence is forbidden and pathologized in favor of use in moderation and ‘all foods fit’ (45)

## Discussion

The literature consistently shows that populations with LCBE also show the characteristics of addiction to alcohol and to UPFs. These include altered reward functions in dopamine, opioid, and cannabinoid pathways as well as stress sensitivity and cognitive impairment. There is also substantial evidence for the role of excessive consumption of addictive UPFs in LCBE, regardless of diagnosis. However, the treatment of binge-type eating disorders characterized by LCBE, such as BED, typically does not include protocols for abstinence from UPFs that can have the kind of psychoactive properties that can cause these neuro-alterations. In a recent prospective, naturalistic, community-based study of individuals with BED, full remission was elusive, occurring in 46% at 5 years, and relapse was common. Specifically, the median time to remission exceeded 60 months, while the median time to relapse was 30 months. However, the percentage of individuals with BED in the community who received treatment for their eating disorder was not reported (107). Even in patients who have received standard inpatient treatment for BED or BN, approximately one-third still were found to meet criteria for an ED 12 years later (108). Another long-term study of patients with BED who took part in a clinical psychotherapy trial showed that a substantial minority of patients (23-48%) either had not responded or relapsed at 12-year follow-up (109).

This inconsistency between the evidence that UPF could be an important contributor to LCBE versus treatment recommendations for reducing or abstaining from UPFs is also found in descriptions of treatment. For example, Wu et al. write that the current standard of care for BED involves psychotherapy, pharmacotherapy, and the management of comorbid conditions, with nutritional rehabilitation reserved for severe cases of anorexia nervosa. The paper goes on to note that unfortunately, many patients often fail to respond, leaving a concerning treatment gap between the current and requisite treatments for EDs (110). It is notable that recommendations to reduce or abstain from UPFs appears nowhere in the major guidelines for the treatment of EDs, including those developed by dieticians (7, 111). Instead, the myth that “all foods fit” all of the time for all types of EDs has persisted despite the lack of supporting data (45).

Further, calls for future research include many topics but not reduction of nor abstinence from psychoactive UPFs (112). In recommendations for future ED research, Hower at al. describe the need to examine predictors of outcomes, biological/neuropsychological techniques, a focus on severe anorexia, a risk calculator, biological and neurological markers, timepoints during which markers begin to emerge, recovery criteria, standardized assessments, lived experience narratives, timelines for recovery in different areas, nuances of the mentorship role, and social media use in at-risk populations (113).

There is no mention in either the Wu or Hower papers of the role of UPFs in the etiology of binge-type EDs, their use by patients with LCBE, their role in the creation of addictive neuro-alterations and loss of control, nor reduction of, or abstinence from, UPFs as a treatment approach.

The absence of any mention of reduction of or abstinence from UPFs is puzzling because in addition to the evidence for the role of abstinence in the restoration of control in SUD treatment, there is also ample justification for reduction in or elimination of UPFs due to well-established consequences of UPF use (114). There is no mention of the consequences of training patients with LCBE to consume UPFs, such as the effect of UPFs on systemic impairment of cell function (115), epidemics of diet-related diseases including mental illnesses (114), nor the 1.7 million Americans who died from diet-related diseases in 2020 (116).

Gearhardt and DiFeliceantonio describe the dangers of overlooking the addictive properties of UPFs by pointing to missed addictive properties of tobacco and resulting failure to regulate, which led to epidemics of severe consequences (112). They go on to point out that SUD recovery approaches focused on abstinence from UPFs are commonly used on Overeaters Anonymous (OA) 12-step fellowships with approximately 6500 weekly meetings worldwide. Food addiction recovery 12 Step fellowships describe abstinence from various UPFs as an essential prerequisite for reversing loss of control and establishing normal eating (104, 105). While there have not been any controlled trials testing the principles of OA or other 12-step approaches for EDS (117), harm reduction and/or abstinence treatment protocols have begun to be tested in clinical populations (46, 47, 81).

There is strong evidence for the benefits of both abstinence and harm reduction in the treatment of SUDs. Abstinence from alcohol has been shown to result in the best long-term control over drinking (118). Attempts to use alcohol socially have been shown to lead to a return to loss of control. Abstinence for AUD does not necessarily mean that abstinence will work for UPFA. This remains to be seen in on-going research (46). However, the harm reduction approach has been shown to be helpful (81). Thus the “all foods fit” approach used in LCBE appears to run counter to evidence that moderate use of UPFs could lead to loss of control.

What follows are five possible explanations for the inconsistency between SUD symptomology and ‘moderate use’ treatment in LCBE populations.

1. Brewerton and colleagues offer several possible explanations for the inconsistency between SUD and binge-type ED treatment (45). Individualized nutritional treatment plans may be quite challenging to employ and oversee in therapeutic settings. Maintaining a simpler “all foods fit” approach in which staff do not have to individualize nutritional approaches for patients with UPFA can be self-serving in that the clinical work of supporting patients who may be triggered by peers with different nourishment plans can be avoided. In addition, ED treatment programs may not recruit staff with the expertise needed to customize food plan (45). Patients with LCBE are typically treated right alongside patients with AN-R and are given similar food plans that are meant to primarily counter caloric restriction, which has long been thought to be a key driving factor in all EDs ever since the “transdiagnostic” theory of EDs was published (119). Specifically, Fairburn and colleagues stated, “binge eating is largely a product of the particular way that these patients attempt to restrict their eating.” Little of no credence has been given to the hedonic, addicting aspects of UPFs in most ED programs.

2. Advocating abstinence from UPFs could also be framed as a threat to UPF markets. As noted, EDs are estimated to occur in 17% of the worldwide population (1), while global estimates of UPFA are 15-20% (26, 120, 121). However, estimates are that most EDs are undiagnosed (122), as is UPFA, suggesting higher numbers of Americans suffer from LCBE and other types of disordered eating, such as “grazing” (123, 124) and would benefit from UPF abstinence and/or reduction. The UPF industry could be fighting through its dietitians to avoid losing that market.

Further, awareness of relief from diet-related diseases that comes with abstinence from UPFs could spread from LCBE populations to the 83% of Americans with overweight and obesity (125). In this scenario, the UPF market could collapse. Americans eat on average 3600 calories per day (126) of which 73% are ultra-processed (127).

3. A third possible explanation for avoiding abstinence protocols in recovery from LCBE is the fear that withdrawing from a UPF could lead to bingeing. However, it has been shown that it can take years to establish consistent abstinence in recovery from SUD (103). Indeed, Koob and Volkow describe the nature of addicted neurocircuitry that supports the idea that lapsing in early recovery is the rule rather than the exception (128). In alcohol recovery, four years has been shown to be needed to achieve consistent abstinence (129). It has also been shown that restriction does not lead to bingeing (130). Transient bingeing has been shown to occur in early withdrawal from sugar and fat (131), but this early-stage lapsing is time-limited and does not seem to warrant avoiding abstinence or harm reduction protocols altogether.

4. Another possibility is that there is confusion about what is meant by ‘restricting.’ Restricting calories, i.e. not eating enough and setting up fear of famine (132) is dangerous. Dieting and fasting have been shown to precede the development of eating disorders (133, 134). This is as opposed to restricting use of UPFs which are associated with disease (114). Pathologizing calorie restriction is justified while pathologizing abstinence from UPFs clearly is not.

5. A final possible explanation for why ED practitioners resist abstinence in treatment of binge eating is that abstinence from UPFs may be beyond the capabilities of LCBE treatment as it is presently structured. The severity of the addiction in LCBE patients is routinely missed and therefore the requisite high level of care is not provided. Research suggests that a majority of Americans might be manifesting enough of the DSM-5 SUD criteria to meet the threshold for severe addiction if the criteria for AUD or other SUD were adapted to loss of control over UPFs. Manifestation of six out of the 11 SUD criteria is the threshold for severity (6). A majority of Americans may be manifesting six criteria including 1) unintended use among the 82% of Americans suffering from overweight, obesity, or severe obesity (125), 2) failure to cut back among the 80% of people who regain lost weight (135), 3) cravings shown to increase with higher BMI (136), 4) use in spite of consequences among the 93% of Americans with a diet-related diagnosis (137), 5) tolerance or progression shown in the general population by an increase in percentage of processed food use among American adults from 67% in 2001 to 72% in 2018 (138), and 6) withdrawal among 70% of people with obesity reporting eating in the absence of hunger (withdrawal avoidance) in a two-week period (139).

Thus, when ED practitioners back away from abstinence or harm reduction strategies because of observations of bingeing in response to using such protocols, it raises the possibility that the bingeing could be due to inadequate support. Severe AUD is treated by various levels of support from residential to intensive outpatient to daily 12 Step support, where harm reduction or abstinence skills can be developed in protected environments free from availability and cueing. This level of protection from UPF cues and availability may not be routinely offered in LCBE treatment.

As shown above, reaching the severity threshold suggests that a treatment match for UPFA would be a high level of care. There are additional reasons to believe that a high level of care is indicated for UPFA. Thus, typical LCBE outpatient care may also be inadequate because of the seriousness of complications associated with UPFA. UPFA may be deeply seated because of intense cueing (140), increased sensitivity to cueing (141, 142), polysubstance use patterns (143), the highly addictive properties of sugar (144), very young age of onset (145), and the drive to conform to social circles that eat UPFs (146, 147). In light of this evidence, it is reasonable to expect that it could take years of neuro-conditioning and skill-building to maintain a healthy level of ultra-processed food abstinence. Cue avoidance, stress management, emotional stability, food production, and relationship skills are among the many skills needed to eat differently from mainstream culture while maintaining a stable, non-craving brain.

Currently, abstinence from addictive UPFs is not an accepted modality for treatment of binge-type EDs. Although dietitians may be hampered by their close association with the UPF industry (148, 149), other practitioners such as therapists, doctors, nurses, and nutritionists may be more open to training in managing complex withdrawal and building skills for abstinence. This gap in treatment raises hope for improved outcomes by offering the high level of support needed to achieve abstinence in cases of severe addiction to UPFs.

## Conclusion

Binge-type EDs are typically treated with food plans that contain UPFs in spite of evidence that UPFs are addictive and that these patients consistently suffer from addictive neuroadaptations including hyperactive reward, impaired cognitive function, and stress reactivity. Low success rates using an ‘all foods fit’ approach point to the need to consider significantly decreased exposure to, or abstinence from, UPFs in their treatment. Possible explanations are offered for why reduction of or abstinence from UPFs is not used in the treatment of binge-type EDs. Research shows that LCBE within the context of UPFA is typically severe and more complicated which suggests that current levels of support could be too low for success in achieving beneficial long-term abstinence from UPFs.

## Data Availability

The original contributions presented in the study are included in the article/supplementary material. Further inquiries can be directed to the corresponding author.
